# Adolescent Engagement in a Binge-Eating Behavioral Health Intervention: Influence of Perceptions of Physical Appearance and Locus of Control

**DOI:** 10.3390/children8020102

**Published:** 2021-02-03

**Authors:** Rebecca C. Kamody, Idia B. Thurston, E. Thomaseo Burton

**Affiliations:** 1 Yale Child Study Center, New Haven, CT 06519, USA; rebecca.kamody@yale.edu; 2 Department of Psychological and Brain Sciences, College of Liberal Arts, Texas A&M University, College Station, TX 77843, USA; idiathurston@tamu.edu; 3 Texas A&M Health Science Center, Department of Health Promotion and Community Health Sciences, School of Public Health, College Station, TX 77843, USA; 4 Department of Pediatrics, College of Medicine, University of Tennessee Health Science Center, Memphis, TN 38103, USA; 5 Children’s Foundation Research Institute, Le Bonheur Children’s Hospital, Memphis, TN 38103, USA

**Keywords:** adolescent, obesity, binge-eating, emotional eating, body image

## Abstract

Traditional weight management approaches focused solely on weight loss as a measure of success may lead youth to internalize negative beliefs about their appearance, and feel they have little control over their health. We examined how perceptions of appearance and health-related locus of control (HRLOC) influenced engagement and outcomes in a behavioral health intervention for binge eating. Thirty adolescents aged 14–18 years completed measures of self-perception, HRLOC, and eating behaviors. Half (*n* = 15) completed baseline assessments only, while the other half participated in a 10-week intervention targeting dysregulated eating behaviors. Analyses revealed negative perceptions of physical appearance and internal HRLOC were higher at baseline among youth who completed the intervention compared to those who completed baseline assessments only. Among those completing the intervention, however, greater internal HRLOC and more positive perception of physical appearance at baseline was associated with greater reduction in objective binge episodes and emotional eating post-intervention. Findings of the present study suggest that while having a more negative perception of one’s appearance may initially motivate youth to participate in weight-related interventions, such perceptions can actually lead to poorer health outcomes, and further supports the extant literature on the benefits of interventions that engender positive body image.

## 1. Introduction

Loss of control eating is well-established as comorbid with obesity in youth, and has been identified as a possibly salient treatment target for both adverse physical and mental health outcomes [1,2,3,4]. Despite being a potentially mutable target for optimal health change among youth [5], addressing loss of control disordered eating among youth is complex. It can influence both anthropometric measures as well as cognitive and emotional states [6], given the guilt and shame associated with such episodes [7]. Historically, many interventions for loss of control eating behaviors have focused on weight loss as a measure of health and success. So termed weight-normative approaches tend to emphasize personal responsibility for weight management and support the belief that weight loss is key to improving most health issues [8]. This style of intervention may contribute to the internalization of negative beliefs about weight and physical appearance, and lead to decreased feelings of control of one’s health (i.e., health-related locus of control (HRLOC)). Overemphasis of shape/weight on one’s self-value has been found to be related to increased weight and more negative perceptions of one’s health [9], and thus may negatively impact engagement and response to intervention, subsequently resulting in long-term impacts on one’s health trajectory.

Discrimination based on one’s weight, known as weight stigma, can have detrimental effects on both physical and mental health outcomes [10,11]. The internalization of these biases may perpetuate the erroneous view that elevated weight is the sole responsibility of the individual, and that the inability to lose weight is their fault due to lack of motivation or laziness [12,13,14]. While the goal of these weight-normative approaches is to promote health and assist with weight loss, their stigmatizing nature can actually lead youth to perceive limited control over their health (i.e., affecting HRLOC), and subsequently result in poorer health outcomes, including weight gain [15]. Furthermore, weight-normative approaches may lead to negative affect and a sense of invalidation, thereby increasing the likelihood of youth seeking comfort in food to soothe and regulate these emotional experiences [16].

Both excess weight and loss of control eating behaviors are complex health concerns influenced by genetic and environmental factors, many of which are not within the control of the individual [8]. Thus, intervention approaches that promote positive self-image and help youth gain control of their eating behaviors, through non-stigmatizing approaches, are of the utmost importance [17,18]. Specifically, interventions focused less on weight loss as the goal, and more so on acceptance and skill acquisition to promote regulation of food intake, may hold promise [19,20,21].

The purpose of the present study was to conduct a set of secondary analyses among adolescents who consented to participate in an outpatient condensed dialectical behavior therapy (DBT) skills group-based intervention, developed to address emotional overeating and binge-eating behaviors [19,20]. The intervention occurred over 10 weeks, and involved a weekly skills group-only intervention, in which select DBT skills (see [19,20] for additional details) were taught by co-leaders in the context of emotional-driven overeating behaviors. The intervention focused on providing adaptive coping skills to replace emotionally-driven overeating behaviors. Both perception of physical appearance and HRLOC were examined as potential factors related to youth engagement in the intervention following baseline assessment. Given the extant literature on the detrimental impact of negative self-perceptions on one’s sense of control over one’s health [8,12,13,15], we hypothesized that youth who completed the intervention would have more positive perceptions of their physical appearance and greater sense of internal HRLOC compared to youth who ended the intervention early.

## 2. Materials and Methods

After obtaining approval from the Institutional Review Board at The University of Tennessee Health Science Center, youth aged 14–18 years were screened for binge-eating and emotional overeating behaviors at three hospital-based pediatric clinics in the Mid-South United States. Youth who endorsed binge-eating symptoms, based on *Diagnostic and Statistical Manual of Mental Disorders, Fifth Edition (DSM-5)* criteria [7], were invited to participate in a DBT skills intervention targeting emotionally-driven overeating behaviors [20]. Exclusion criteria included diagnoses of Intellectual Disability and lack of parental consent. As a skills-based intervention adjunctive to clinical care, youth were not excluded based on appetitive-relevant medication nor medical conditions that could impact eating, given that youth with such conditions could still benefit from learning these skills. At initial baseline assessment, informed consent was obtained from all participants (*n* = 30; *M*_age_ = 15.44; 93% Black; 67% Female; *M*_BMI Z-Score_ = 2.30). Youth completed the following measures: Self-Perception Profiles for Adolescents (SPPA) Physical Appearance Scale, which utilized descriptive statements and ratings scales in order to assess self-evaluation of importance of physical appearance [22]; Multidimensional Health Locus of Control Scale (MHLC) [23], which assessed participants’ belief about what influenced their health (including internal, chance, and others); Emotional Eating Scale (EES) [24], which assessed frequency of emotional eating behaviors; and reported frequency of monthly objective binge episodes (OBEs) on the Eating Disorder Examination Questionnaire [25]. The intervention yielded a 50% attrition rate, with 15 youth ending the intervention early. All group members lost to attrition (considered Non-Completers) was due to their choice not to continue. Those considered Non-Completers all discontinued by the third session (descriptive group differences between Intervention Completers and Non-Completers are reported elsewhere) [19].

## 3. Results

**Figure 1:**
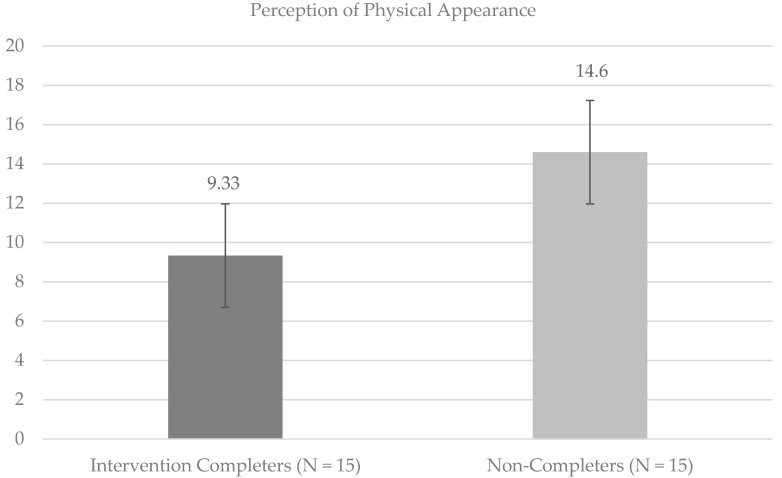
Baseline means and standard errors for perceptions of physical appearance. Note. On the SPPA PA scales, lower scores are indicative of more negative perceptions of physical appearance, and higher scores indicative of positive perception of physical appearance.

**Figure 2:**
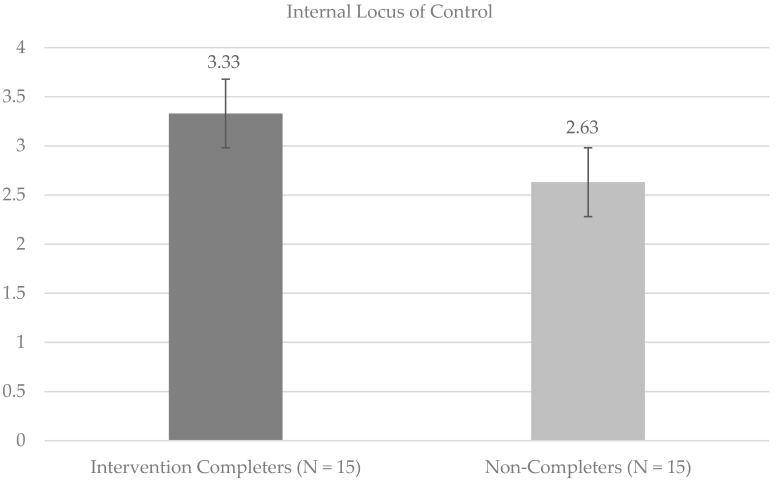
Baseline means and standard errors for perceptions of internal locus of control.

Independent samples *t*-tests were used to compare perceptions of physical appearance and sense of internal HRLOC among Intervention Completers and Non-Completers. Significant differences on the SPPA Physical Appearance (SPPA PA) subscale suggested more negative perceptions of physical appearance at baseline (*t*(28) = −3.48, *p* < 0.05) among Intervention Completers compared to Non-Completers (Cohen’s *d* = 1.31; large effect size). Internal HRLOC (*t*(28) = 2.15, *p* < 0.05) was significantly higher among Intervention Completers compared to Non-Completers at baseline (Cohen’s *d* = 0.81; large effect size). Figure 1 and Figure 2 provide mean values for the SPPA PA and MHLC Internal subscales, respectively. Post hoc Shapiro–Wilk tests of normality were run, and supported the conclusion that the data were normally distributed for the Intervention Completers on the SPPA PA (statistic = 0.97, *p* = 0.83) and internal HRLOC (statistic = 0.96, *p* = 0.64) scales, as well as for Non-Completers on the SPPA PA (statistic = 0.89, *p* = 0.07) and internal HRLOC (statistic = 0.92, *p* = 0.17) scales.

Among Intervention Completers only, bivariate correlations were run between the SPPA PA and MHLC Internal subscales with change scores in OBEs and emotional eating (based on the EES) from baseline to post-intervention. Higher internal HRLOC and more positive perceptions of appearance at baseline were both associated with greater reduction in OBEs and emotional eating. Mean scores and correlations are presented in Table 1.

## 4. Discussion

The findings of the current study support the extensive literature on the harmful impact of negative internalized views of physical appearance on health outcomes [8,11,12,13,15]. Contrary to the original hypothesis, the present findings suggest that having a more negative view of one’s physical appearance may initially motivate youth to participate in behavioral health interventions for weight and eating behaviors. It is very important to note, however, that these negative self-views may actually lead to poorer outcomes once youth are engaged in treatment. While the dissonance between one’s current and idealized physical appearance may serve to initially prompt efforts to change, the long-term effects of this negative internalization of one’s appearance is likely to in fact be detrimental to long-term weight and health outcomes [8,12,13,15].

This study presents novel associations among a unique population of predominantly Black youth endorsing binge-eating behaviors and emotional overeating, who initially agreed to participate in a behavioral health intervention for these behaviors. Although the 50% attrition rate may be viewed as a weakness of the original study [19], we used this potential weakness as an opportunity to conduct secondary analyses of two equally-sized samples to better understand factors that may have contributed to engagement and attrition. While the small sample size of the present report may also be viewed as a potential study limitation, we contend that the small size of the sample for these secondary analyses allows for strong inferential validity of the present study [26]. Moreover, it allows for a more systematic investigation of the functional relationships among these variables.

Among adults, the benefit of multiple approaches (e.g., a structured physical activity intervention in addition to cognitive-behavioral therapeutic approaches) has been established in the treatment of binge eating disorder (BED) symptoms [27]. However, the development and implementation of behavioral health interventions that are sensitive to the unique needs of adolescents is of paramount importance. While weight-normative approaches can motivate adolescents to engage in interventions to improve their health, subsequent internalized stigmatizing perceptions may deter progress in treatment once youth are engaged in treatment. Thus, the utilization of strength-based interventions by clinicians working with youth presenting with loss of control eating, that engender positive body image and bolster internal HRLOC, may be more effective at targeting dysregulated eating in youth. Clinicians and medical providers working with youth with elevated weight and disordered eating presentations are recommended to consider these additional factors when collaboratively working with youth on eating, weight, and health goals.

Findings align with the theoretical conceptualizations of the detrimental effect of negative self-views on behavioral health outcomes and indicate a need for larger scale quantitative studies that further assess weight-inclusive approaches to eating and weight interventions for adolescents. Future studies should continue to critically evaluate the roles of treatment approaches and individual characteristics on intervention engagement among youth, in order to promote the most optimal interventions for adolescent health.

## Figures and Tables

**Table 1:** Bivariate correlations among Intervention Completers.

	Perception of Physical Appearance	Internal Locus of Control	Reduction in Objective Binge Episodes	Reduction in Emotional Eating
Perception of Physical Appearance (α = 0.85)	9.33 (2.99)	0.39	0.54 *	0.77 **
Internal Locus of Control (α = 0.77)	-	3.33 (0.91)	0.64 *	0.69 **
Reduction in Objective Binge Episodes (α = 0.83)	-	-	1.54 (1.06)	0.80 **
Reduction in Emotional Eating (α = 0.96)	-	-	*-*	2.93 (0.13)

Note. Diagonal of table provides means (and standard deviations). Alpha for reduction in OBEs represents the alpha of the EDE-Q; alpha for reduction in emotional eating represents the alpha of EES. * *p* < 0.05; ** *p* < 0.01.

## Data Availability

The data presented in this study are available upon reasonable request from the corresponding author.
